# Dietary Escitalopram Reduces Movement Variability and Enhances Behavioral Predictability in *Drosophila melanogaster*

**DOI:** 10.3390/biology15010051

**Published:** 2025-12-28

**Authors:** Vadims Kolbjonoks, Sergejs Popovs, Ronalds Krams, Giedrius Trakimas, Māris Munkevics, Tatjana Krama, Markus J. Rantala, Jorge Contreras-Garduño, André Rodrigues de Souza, Colton B. Adams, Priit Jõers, Indrikis Krams

**Affiliations:** 1 Department of Technology, Institute of Life Sciences and Technology, Daugavpils University, 5401 Daugavpils, Latvia; vadims.kolbjonoks@du.lv; 2 Department of Biodiversity, Institute of Life Sciences and Technology, 5401 Daugavpils, Latvia; sergey.p@email.com (S.P.); ronalds.krams@gmail.com (R.K.); giedrius.trakimas@gf.vu.lt (G.T.); marismunkevics@gmail.com (M.M.); tatjana.krama@du.lv (T.K.); colton.adams@fulbrightmail.org (C.B.A.); 3 Latvian Biomedical Research and Study Centre, 1067 Riga, Latvia; 4 Institute of Food Safety, Animal Health and Environment “BIOR”, 1076 Riga, Latvia; 5 Institute of Biosciences, Life Sciences Center, Vilnius University, 10257 Vilnius, Lithuania; 6 Department of Ecology, Faculty of Medicine and Life Sciences, University of Latvia, 1048 Riga, Latvia; 7 Chair of Plant Health, Institute of Agricultural and Environmental Sciences, Estonian University of Life Sciences, 51014 Tartu, Estonia; 8 Department of Biology, University of Turku, 20014 Turku, Finland; mjranta@utu.fi; 9 Laboratorio de Ecología Evolutiva, Escuela Nacional de Estudios Superiores, Unidad Morelia, Universidad Nacional Autónoma de México, Morelia 58190, Mexico; jcg@enesmorelia.unam.mx; 10 Departamento de Biologia Geral, Universidade Federal de Viçosa, Viçosa 36570-900, Brazil; andrebioufjf@gmail.com; 11 Department of Ecology and Evolutionary Biology, University of Tennessee, Knoxville, TN 37996, USA; 12 Department of Psychology, University of Tennessee, Knoxville, TN 37996, USA; 13 Institute of Molecular and Cell Biology, University of Tartu, 51010 Tartu, Estonia; priit.joers@ut.ee

**Keywords:** animal personality, behavioral individuality, *Drosophila melanogaster*, escitalopram, behavioral lateralization, serotonin, turning behavior

## Abstract

Behavioral individuality, often termed animal personality, reflects consistent patterns of behavioral variability across individuals. In fruit flies (*Drosophila melanogaster*), pharmacological and dietary manipulations affecting neuromodulatory systems have been shown to alter behavior, but their effects on behavioral predictability remain incompletely understood. Here, we investigated whether developmental dietary exposure to tryptophan (a serotonin precursor) or escitalopram (a selective serotonin reuptake inhibitor, SSRI) is associated with changes in lateralized turning behavior. Flies were reared from larval stages on supplemented media and tested in a Y-maze assay to assess movement predictability. Flies exposed to escitalopram displayed significantly reduced behavioral variability compared to controls, indicated by a lower median absolute deviation (MAD) of turning behavior, whereas tryptophan supplementation did not significantly affect variability. Because both compounds were tested at a single dietary dose and serotonergic activity was not directly measured, these findings should be interpreted as dose-specific behavioral effects rather than evidence of altered serotonergic tone or mechanism. Our results demonstrate that chronic developmental exposure to escitalopram is associated with increased behavioral predictability in fruit flies, highlighting the utility of high-throughput behavioral assays for detecting subtle pharmacologically induced changes in individual variability. Future studies incorporating dose–response designs and physiological validation will be required to establish underlying mechanisms.

## Simple Summary

Animals often differ consistently from one another in how they behave, a phenomenon known as behavioral individuality. Such differences can be important for how animals cope with changing or risky environments. In this study, we examined whether long-term dietary exposure to two commonly used compounds, tryptophan and escitalopram, is associated with differences in behavioral variability in fruit flies (*Drosophila melanogaster*). Flies were reared on food containing either tryptophan, escitalopram, or control food, and their movement decisions were later measured in a Y-shaped maze. We found that flies exposed to escitalopram showed reduced variation among individuals in turning behavior, resulting in more predictable movement patterns, whereas tryptophan exposure was not associated with consistent changes in behavioral variability. These findings indicate that chronic exposure to escitalopram during development is associated with altered patterns of behavioral individuality in fruit flies.

## 1. Introduction

Behavioral variability and individuality are increasingly recognized as fundamental traits in animal populations, enabling flexible responses to dynamic environments [1]. The emergence, maintenance, and flexibility of behavior are shaped by interacting physiological and environmental factors. For example, in *Drosophila melanogaster*, individual differences in turning bias and phototaxis persist even among genetically identical flies reared under uniform conditions [2,3,4]. Such stable tendencies, often described as animal personality [4,5,6], reflect not only mean behavioral tendencies but also the structured variability of responses [7]. Personality research therefore emphasizes the role of neurophysiological processes in generating individual behavioral outcomes, particularly in model systems that allow experimental access to neural pathways.

Serotonin (5-HT) is a key neuromodulator implicated in mood regulation, behavioral flexibility, and stress responsiveness across taxa [8,9]. In *Drosophila*, manipulations that affect monoaminergic systems—including serotonergic pathways—have been associated with changes in behavioral variability and predictability, while central complex circuits maintain stable lateralized behaviors [4]. Developmental exposure to predation risk has been shown to alter both behavior and monoaminergic signaling in flies [10,11], suggesting that neuromodulatory systems may interact with environmental inputs to shape behavioral outcomes, although the specific mechanisms remain unresolved. Together, these findings point to serotonergic systems as plausible contributors to behavioral predictability, without implying a single causal pathway.

Recent studies in insects and mammals [12] have reported that pharmacological manipulations affecting serotonergic signaling can be associated with reduced behavioral variability, a key trait linked to personality. Krams et al. (2018) [13] demonstrated that selective serotonin reuptake inhibitors (SSRIs) altered behavioral consistency in crickets (*Gryllus integer*), particularly along the coping-style axis, with effects depending on developmental speed. Importantly, these antidepressant treatments reduced behavioral variability without producing uniform directional changes in behavior, highlighting the context-dependent and non-linear nature of neuromodulatory effects [13]. Similarly, Trakimas et al. (2019) [14] linked developmental speed to physiological stress resilience in crickets, suggesting that early life-history strategies influence adult behavioral predictability. Collectively, these studies motivate further examination of how developmental exposure to neuromodulator-related dietary or pharmacological treatments relates to individual behavioral variability in genetically tractable systems.

In this study, we tested whether developmental dietary exposure to tryptophan (a serotonin precursor) or escitalopram (a selective serotonin reuptake inhibitor, SSRI) is associated with altered behavioral predictability of turning behavior in adult flies. Rather than inferring specific neurochemical mechanisms, we asked whether these two commonly used serotonergic manipulations differ in their effects on movement variability. Based on previous behavioral studies, we predicted that escitalopram exposure during development would be associated with reduced behavioral variability, potentially constraining the expression of personality-related traits in *D. melanogaster* [13,14]. Because serotonergic activity was not directly measured and only a single dietary dose was tested, these predictions are framed at the behavioral level rather than as mechanistic hypotheses. To our knowledge, this is the first study to experimentally examine whether developmental exposure to an SSRI is associated with altered movement predictability in insects.

Understanding how developmental pharmacological exposure relates to behavioral predictability in a simple model organism may provide a behavioral framework for future mechanistic studies, including those relevant to affective blunting or cognitive rigidity in human mood disorders. Because behavioral variability itself can differ across *Drosophila* genotypes [3], we focused on a single inbred line to minimize genetic heterogeneity. To further control confounding factors, we tested only males and reared flies under identical environmental conditions. Thus, experimental groups differed only in dietary treatment (tryptophan, escitalopram, or control) and naturally arising inter-individual differences, enabling us to isolate treatment-associated effects on behavioral variability [15]. Because monoaminergic pathways are developmentally intertwined in *Drosophila*, sharing enzymes and partially overlapping neuronal populations, our aim was not to disentangle dopamine- and serotonin-specific mechanisms. Instead, we compared a precursor-based manipulation (tryptophan/5-HTP) with a relatively serotonin-selective reuptake blockade (escitalopram) as complementary developmental perturbations of monoaminergic systems, recognizing that downstream effects may not be serotonin-specific [16].

Importantly, beyond mean behavioral tendencies, the degree of unpredictability in behavior itself can have direct fitness consequences. Previous work has demonstrated that *Drosophila* development is highly sensitive to environmental stressors, including predation risk, which produces lasting physiological and behavioral changes in the adult phenotype [17]. In particular, our earlier studies showed that flies exhibiting greater turning variability are less likely to be captured by spiders, demonstrating that intragenotypic variation is not merely statistical noise but an adaptive trait influencing antipredator performance and memory [11,17,18,19]. These findings align with the broader concept of protean escape behavior, whereby erratic and unpredictable movement reduces predator capture success across taxa [20,21]. Together, this literature highlights behavioral variability as a selectable trait that can be shaped by both ecological and developmental perturbations, reinforcing the need to examine variability explicitly rather than focusing solely on mean behavioral effects.

## 2. Materials and Methods

### 2.1. Animals and Experimental Groups

Wild-type *D. melanogaster* (Oregon-R-modENCODE #25211, Bloomington Drosophila Stock Center, Bloomington, IN, USA) were used in this study. The line was inbred (full-sib matings) for 10 generations prior to behavioral testing. To minimize genetic variation, only males were used, and all individuals were reared under standardized laboratory conditions to reduce environmental sources of behavioral variation. Thus, experimental groups differed only in the chemical treatment of their food (tryptophan, escitalopram, or control) and in naturally arising inter-individual differences during development.

Flies were reared under standard laboratory conditions (21 °C, 12:12 h light–dark cycle, 60% relative humidity) in January 2023. From the larval stage onward, flies were assigned to one of three groups: (1) Control, reared on standard fly medium adapted from the Cold Spring Harbor Protocols ([22]; 100 mL water mixed with 4 g dextrose, 7 g cornmeal, 0.9 g agar, and 2 g deactivated yeast); (2) Tryptophan, reared on the same medium supplemented with 5-hydroxy-L-tryptophan (5-HTP; Sigma Aldrich H9772) at a final concentration of 1 mg/mL [23]; and (3) Escitalopram, reared on medium supplemented with escitalopram oxalate (Sigma Aldrich 219861-08-2) at a final concentration of 10 µM. A dietary escitalopram concentration of 10 µM falls within the range shown to alter serotonin clearance kinetics in bath-applied larval CNS preparations [24]. However, Dunham et al. (2024) [25] demonstrated dietary reuptake inhibition only at ≥1 mM, and therefore, the effects of 10 µM feeding on whole-brain serotonin levels remain unknown. Accordingly, we interpret our results as behavioral consequences of chronic low-dose SSRI exposure rather than as direct evidence of increased serotonin concentration. During pilot trials, 5-HTP and escitalopram were dissolved in deionized water together with Blue FCF dye (Acros Organics A0373695, Thermo Fisher Scientific, Waltham, MA, USA) and incorporated into the food medium. Blue staining was observed in larvae across treatment groups, confirming ingestion of the supplemented diet [23]. We used escitalopram because it is highly selective for the serotonin transporter (SERT), the primary protein responsible for serotonin reuptake, and has demonstrated reliable behavioral and physiological effects in our previous studies with invertebrates [13]. Although other SSRIs are widely used in *Drosophila* research, our selection allowed direct continuity with prior work and minimized interpretive variability related to differences in drug selectivity or off-target activity.

Importantly, both the 5-HTP and escitalopram treatments were administered at a single dietary dose, and the present experiment was not designed as a dose–response study. Thus, all treatment effects reported here should be interpreted as dose-specific outcomes rather than general pharmacological profiles across concentrations.

Since flies with different developmental trajectories may vary significantly in body mass, elemental composition, food intake, fat metabolism, and behavior, only individuals with intermediate developmental speed were used in this study [18]. These flies eclosed on day 12 at 21 ± 1 °C.

### 2.2. Behavioral Testing

Adult flies, those at 3–5 days post-eclosion (SSRI treatment group, *N* = 450; 5-HTP group, *N* = 410; control group, *N* = 425), were tested individually in a custom-built Y-maze arena [4,11,15] to assess spontaneous turning behavior. Flies were put into an array containing 95 individual Y-mazes consisting of three symmetrical arms (each 12 mm long) fabricated from laser-cut acrylic (Figure S1, Supplementary Material). Flies were gently introduced into the maze using an aspirator and allowed to explore freely for two hours under uniform, diffuse illumination (approximately 500 lux) at 22 °C. Each maze arm was identical in appearance and construction to minimize external cues influencing directional choices.

### 2.3. Data Collection and Analysis

Behavioral trajectories were recorded with high-definition cameras and tracked using EthoVision XT v.15.0 software (Noldus Information Technology, Wageningen, The Netherlands). Turning direction (left or right) was automatically extracted for each decision point. To quantify the extent of variation in turn bias (behavioral predictability), we calculated the median absolute deviation (MAD) of turning choices (coded as 1 for right turns and 0 for left turns) for each fly. A higher MAD indicates greater variability (lower predictability), while a lower MAD indicates more stereotyped behavior (greater predictability). MAD was chosen as our metric for variability because it is well characterized, nonparametric, and weights data points equally.

Permutation tests (10,000 iterations) were conducted to compare MAD values between treatment groups. We chose this non-parametric approach because MAD values derived from binary left–right decisions may deviate from normality, and permutation testing does not rely on distributional assumptions. We used MAD as an index of behavioral predictability in individual flies, since our goal was to measure variability in turning behavior rather than central tendency (mean or median). Bootstrapping methods were applied to estimate confidence intervals around group MAD values. As our analyses focused on MAD values with bootstrapped standard errors rather than full distributions of raw turning data, visualization was restricted to barplots with error estimates rather than boxplots or violin plots.

We checked for differences across treatments in turn bias by using Analysis of Variance (ANOVA). We set the proportion of right turns taken as a dependent and treatment as an independent variable. We checked for normality of residuals using the Shapiro–Wilk test, and for homoscedasticity with Levene’s test.

We also compared the number of turns per minute taken by flies across treatments using Kruskal-Wallace test due to data not being normally distributed. As a post hoc, we used pairwise Mann–Whitney U test, with Benjamini & Hochberg for multiple comparisons.

Statistical analyses were performed using R version 4.2.1. Differences were considered statistically significant at *p* < 0.05.

### 2.4. Ethics Approval Statement

The manuscript presents research on animals that do not require ethical approval for their study.

## 3. Results

**Figure 1:**
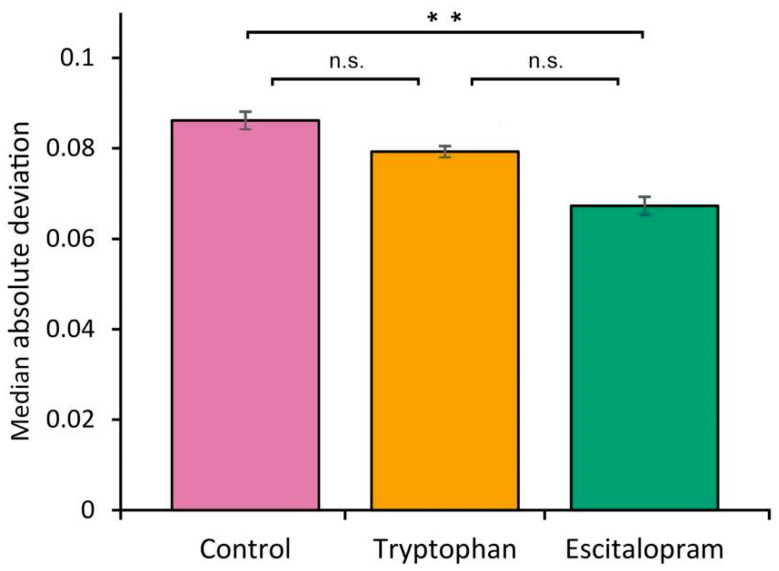
Distribution of turning deviation in *Drosophila melanogaster* (Oregon R strain) as a function of antidepressant escitalopram treatment. Error bars represent the standard error of the mean (SEM). Asterisks indicate significant difference (*p* < 0.002); n.s. = non-significant (*p* > 0.05).

Dietary treatment with escitalopram significantly reduced behavioral variability in turning behavior compared to the control group. Median absolute deviation of turning direction was lower in the escitalopram group than in untreated controls (MAD = 0.067 vs. 0.086; *p* < 0.002), indicating increased predictability. Tryptophan treatment did not significantly alter MAD relative to the control group (MAD = 0.080 vs. 0.086; *p* > 0. 05) and the escitalopram group (MAD = 0.080 vs. 0.067; *p* > 0.05) (Figure 1). These results suggest that escitalopram narrows the range of behavioral responses without shifting mean turning bias, thereby suppressing individual variability.

**Figure 2:**
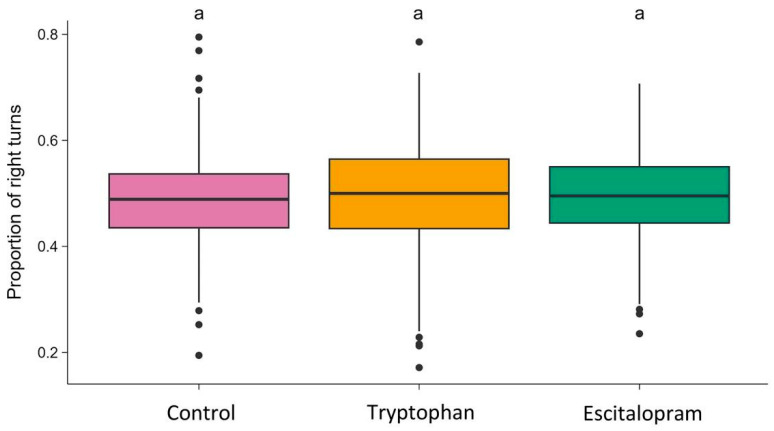
Proportion of right turns in the control, tryptophan-treated, and escitalopram-treated groups. The horizontal line indicates the group median. Dots represent individual observations. Groups sharing the same letter are not significantly different.

The proportion of right turns taken did not differ between groups statistically significantly (one-way ANOVA: F_2,587_ = 0.384; *p* = 0.6811) (Figure 2).

**Figure 3:**
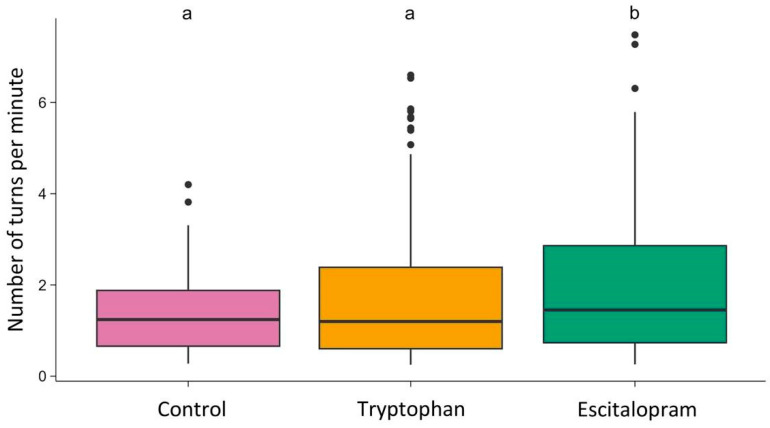
Number of turns per minute in the control, tryptophan-treated, and escitalopram-treated groups. The horizontal line indicates the group median. Dots represent individual observations. Groups sharing the same letter are not significantly different; groups with different letters differ significantly.

The number of turns per minute differed between groups (Kruskal–Wallis H = 8.01; df = 2; *p* = 0.018) (Figure 3). Pairwise comparison revealed that flies treated with escitalopram (median = 1.45; Q1 = 0.73; Q3 = 2.86) took significantly more (Pairwise Mann–Whitney U test with Benjamini & Hochberg correction: *p* = 0.038) turns per minute than flies treated with 5-HTP (median = 1.2; Q1 = 0.6; Q3 = 2.39), and also significantly more (*p* = 0.034) turns than control flies (median = 1.24; Q1 = 0.65; Q3 = 1.88). Control flies and flies treated with 5-HTP did not differ significantly in terms of turns taken per minute.

## 4. Discussion

Our findings show that escitalopram reduced variability (increased predictability) in turning behavior while elevating overall turning activity. Because directional bias did not differ between groups, these effects are unlikely to reflect impaired motor function and instead are consistent with altered decision-making dynamics associated with developmental pharmacological exposure. While escitalopram is classically described as an SSRI, we do not assume direct serotonergic causation, and instead interpret the observed patterns at the behavioral level. This interpretation is consistent with previous studies linking monoaminergic modulation to stabilized behavioral trajectories in invertebrates [4,11]. Several studies have examined chronic developmental pharmacological manipulations affecting monoaminergic systems in *Drosophila*, including long-term treatments spanning larval to adult stages [26], showing that such exposures can influence locomotor behavior and neural function across development. Prior work has further shown that variability in phototactic and turning behavior is itself a consistent, repeatable trait [2,4], supporting its interpretation here as an aspect of animal personality or individuality [27]. We emphasize that all conclusions are restricted to the single dietary doses tested and should not be generalized to dose–response relationships or specific neurochemical mechanisms.

Escitalopram-treated flies performed significantly more turns per minute than both control and 5-HTP–treated flies, whereas the latter two groups did not differ. However, the proportion of right turns did not differ statistically between groups, indicating that escitalopram did not bias directional preference. Thus, escitalopram exposure was associated with increased activity and reduced variability, consistent with a shift toward more stereotyped—but not directionally biased—behavioral output. Such stereotypy does not imply reduced motor capacity but rather reduced variability in action selection. These findings resonate with mammalian studies reporting that chronic SSRI treatment can enhance motor activity while constraining behavioral flexibility [9,12]. Although the tryptophan and escitalopram groups appear visually separated based on SEM bars in Figure 1, SEM overlap is not a test of significance. The statistical comparison relies on replicate-level variation, and the much broader dispersion in the tryptophan group reduces statistical power, resulting in a non-significant contrast.

Our findings align with earlier work on crickets, where SSRIs influenced coping styles and stress reactivity depending on developmental traits [13]. Although the present study did not assess developmental speed directly [18], the parallel outcomes across species suggest that developmental context modulates sensitivity to pharmacological perturbation, rather than pointing to a conserved molecular mechanism. Trakimas et al. (2019) [14] also demonstrated links between life-history speed and stress physiology, which may contribute to why some individuals or species differ in behavioral responsiveness. Comparable outcomes in mammals—for example, altered behavioral responses to fluoxetine in rats with differing baseline neuromodulatory states [12]—support the idea that developmental and physiological background shapes behavioral consequences of pharmacological exposure.

Our results therefore suggest that chronic developmental escitalopram exposure is associated with a reduced range of individual behavioral expression, consistent with decreased behavioral individuality. This contrasts with earlier findings showing that ecological stressors can increase behavioral variability [11,28]. Rather than invoking opposing serotonergic mechanisms, we interpret this contrast as evidence that environmental and pharmacological perturbations can shape variability in opposite directions, depending on timing, intensity, and regulatory feedback. Long-term SSRI exposure is known to elicit neuroadaptive responses in vertebrates, including changes in receptor sensitivity and transporter expression [29,30]. Although such processes may plausibly contribute to the patterns observed here, we do not test these mechanisms directly, and therefore treat these explanations as provisional. As in mammalian systems, prolonged SSRI exposure can influence receptor plasticity and downstream regulation [9,31], but mechanistic inference is limited by the absence of neurochemical or genetic assays in the present study.

Tryptophan supplementation did not significantly affect behavioral variability. This null effect may reflect limited conversion to central monoamines, strong metabolic regulation of precursor availability, or reduced sensitivity of behavioral variability to precursor-based manipulation at the tested dose. Notably, tryptophan did not produce effects opposite to escitalopram but showed only a weak trend in the same direction. This pattern is consistent with evidence that precursor supplementation often produces smaller and more variable behavioral effects than transporter blockade, particularly at low doses. Similar distinctions between precursor and SSRI effects are reported in clinical and animal studies, where SSRIs are more strongly associated with emotional blunting or reduced exploratory variability [12,32].

Our study does not determine how 10 µM dietary escitalopram or 1 mM dietary 5-HTP affect serotonin concentration in vivo. Evidence from Dunham and Venton (2022) [24] shows that 10 µM bath-applied escitalopram alters serotonin clearance kinetics in dissected larval CNS preparations, but such in vitro results cannot be extrapolated to dietary exposure in intact animals. To our knowledge, no published study has quantified neurochemical consequences of dietary escitalopram at sub-millimolar concentrations. Likewise, Majeed et al. (2016) [26] observed serotonin changes only at 5–25 mM dietary 5-HTP, while lower doses—including the 1 mM concentration used here—were not examined. Accordingly, we do not assume serotonergic elevation, and interpret the observed behavioral effects as dose-specific outcomes of chronic low-level neuromodulatory exposure. Future studies incorporating multiple doses and direct neurochemical measurements will be essential to establish causal pathways.

Despite these mechanistic limitations, the behavioral findings themselves are robust. The study includes a large sample size (1285 flies), yielding high statistical power and highly consistent behavioral patterns across independent arenas. Importantly, the Y-maze assay clearly discriminates between control flies and those reared on supplemented diets and distinguishes the behavioral signatures of tryptophan and escitalopram. These results demonstrate that even low-dose developmental dietary manipulations can reliably alter behavioral variability and activity, establishing a strong behavioral foundation for future mechanistic work.

Although serotonin and dopamine are synthesized via distinct biochemical pathways, extensive interaction between monoaminergic systems occurs at the circuit level. Recent studies document dopamine–serotonin co-transmission and receptor-mediated cross-talk [33]. In insects, octopamine plays a major role in arousal and locomotor regulation. While these neuromodulators were not manipulated here, future integrative studies will be important for understanding how multiple systems jointly regulate behavioral variability.

Although this study did not examine depression-like states or experimentally induced stress, prior work has shown that antidepressants can reverse stress-induced behavioral suppression in *Drosophila* [23]. Applying the present behavioral framework to stress-exposed flies would allow direct tests of how pharmacological treatments interact with environmental adversity, offering a high-throughput platform for studying stress-related phenotypes [34].

Finally, because our study relied on pharmacological manipulations rather than genetic controls, our evidence is correlative. The absence of receptor-specific, transporter-based, or neurochemical assays limits mechanistic inference. In addition, we examined only males, despite known sex differences in neuromodulatory function. Future studies should integrate genetic tools (e.g., *Trh*-GAL4, serotonin transporter or receptor mutants), dose–response designs, and sex comparisons to establish causal pathways and generalize the behavioral patterns reported here.

## 5. Conclusions

Developmental dietary exposure to escitalopram was associated with reduced inter-individual variability and increased predictability of turning behavior in *D. melanogaster*, without affecting mean directional bias. In contrast, tryptophan supplementation did not produce significant effects at the tested dose. These findings demonstrate that low-dose developmental SSRI exposure can constrain behavioral variability and highlight behavioral predictability as a sensitive outcome for detecting pharmacologically induced effects in model organisms.

## Data Availability

The dataset presented in this study can be found in the ZENODO online repository: doi:10.5281/zenodo.17035371.

## Supplementary Materials

The following supporting information can be downloaded at: https://www.mdpi.com/article/10.3390/biology15010051/s1, Figure S1: Detail of Y-mazes containing individual flies.
