# Supplementary figures and images for: Dietary Escitalopram Reduces Movement Variability and Enhances Behavioral Predictability in Drosophila melanogaster
Source: Biology (Basel). 2025 Dec 28;15(1):51. doi: 10.3390/biology15010051 (PMC12784902; doi:10.3390/biology15010051)

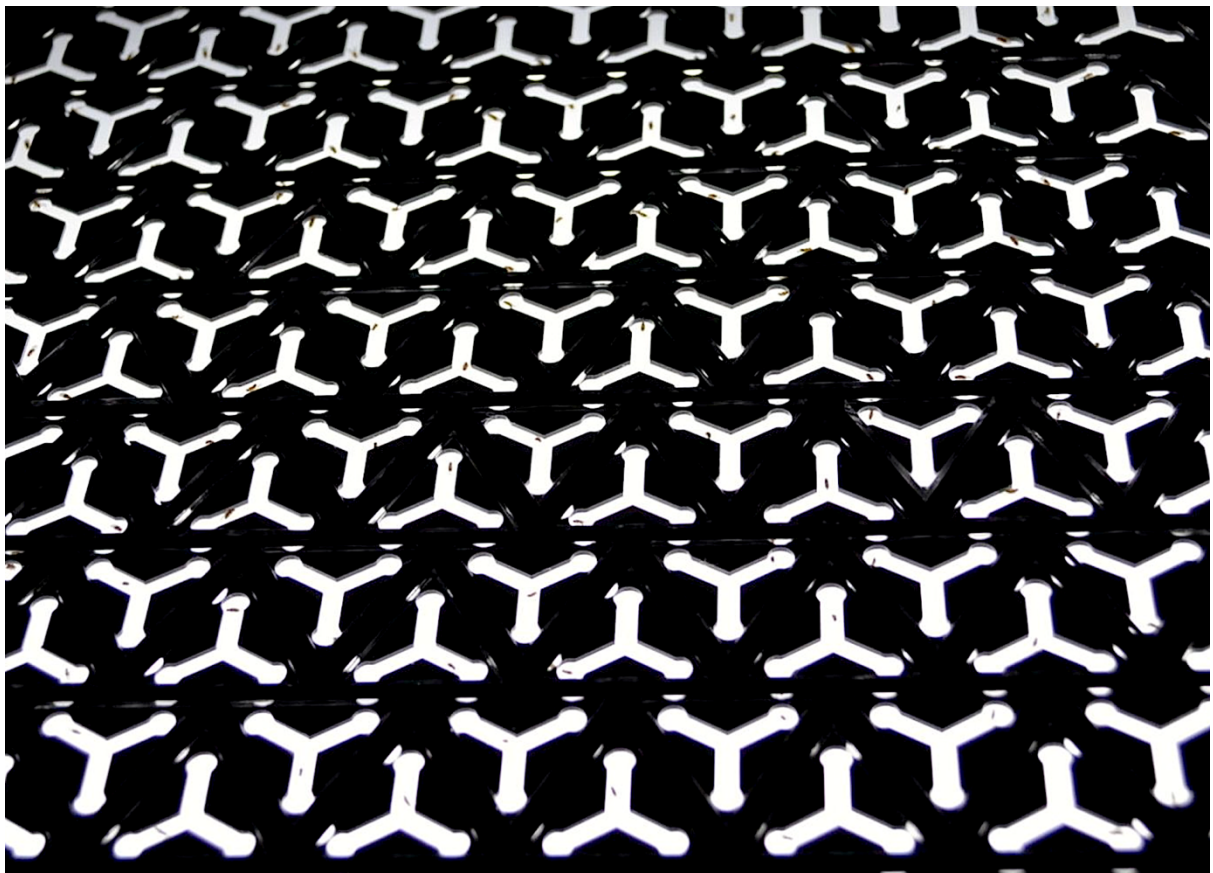

Figure S1. Detail of Y-mazes containing individual flies.

Supplement: Supplementary file 1 [file biology-15-00051-s001.zip › biology-3980822-supplementary.pdf]
